# Caspases in synaptic plasticity

**DOI:** 10.1186/1756-6606-5-15

**Published:** 2012-05-14

**Authors:** Zheng Li, Morgan Sheng

**Affiliations:** 1 Unit on Synapse Development and Plasticity, National Institute of Mental Health, National Institutes of Health, Bethesda, MD 20892, USA; 2 Department of Neuroscience, Genentech Inc, South San Francisco, CA 94080, USA

## Abstract

Caspases are a family of cysteine proteases that play key roles in programmed cell death (apoptosis). Mounting evidence in recent years shows that caspases also have important non-apoptotic functions in multiple cellular processes, such as synaptic plasticity, dendritic development, learning and memory. In this article, we review the studies on the non-apoptotic functions of caspases in neurons, with a focus on their roles in synaptic plasticity, learning and memory and neurodegeneration.

## Introduction

Caspases are a family of cysteine proteases that have a conserved cysteine residue at their active site and cleave after an aspartate residue in their substrates. As key proteolytic enzymes involved in programmed cell death (or apoptosis), caspases are found in a wide range of animals from worms to humans; in mammals, 12 caspases have been identified. Caspases are generally translated as inactive zymogens and activated through proteolytic cleavage. Based on their structure and function, caspases are classified into two groups: initiator caspases and effector caspases. Initiator caspases (caspase-1, -2, -4, -5, -8, -9, -10, -11 and -12) have a long N-terminal prodomain through which they are recruited to specific protein complexes for activation. Once activated, initiator caspases can cleave and activate downstream effector caspases (e.g. caspase-3, -6, -7, -14), which then go on to proteolyze further cellular substrates, of which many examples are now known [1].

**Figure 1:**
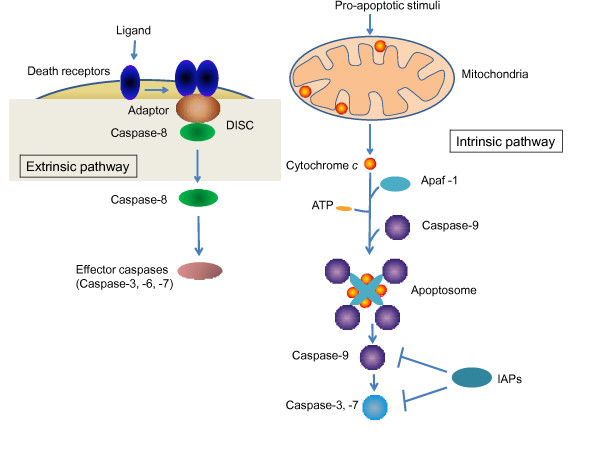
**Extrinsic and intrinsic pathways of apoptosis**. The two major apopotosis pathways are illustrated. The extrinsic pathway is initiated by ligand binding to death receptors on the plasma membrane. The intrinsic pathway is also called the mitochondrial pathway. Both pathways lead to activation of caspases.

Since the discovery of the critical function of the C. elegans caspase ced-3 in programmed cell death [2,3], most members of the caspase family have been demonstrated to be components of apoptotic signaling pathways. The biochemistry and function of these proteases have been predominantly studied in the context of apoptosis. In cells undergoing apoptosis, caspases are activated by two main pathways: the extrinsic pathway and the intrinsic pathway (see Figure 1). The extrinsic pathway is initiated by binding of specific ligands (e.g. tumor necrosis factor alpha [TNFα], Fas ligand, Nerve growth factor [NGF]) to cell surface "death receptors", such as tumor necrosis factor receptor 1 (TNFR1), Fas and nerve growth factor receptor p75NTR [4]. Upon ligand binding, the death receptors multimerize and recruit multiple adaptor molecules to form the death-inducing signaling complex (DISC), which in turn interacts with and activates the initiator caspases [1]. For TNFR1, TNF receptor associated-protein with death domain (TRADD), TNF receptor associated protein 2 (TRAF2), receptor associated protein kinase 1 (RIPK1), cellular inhibitor of apoptosis proteins cIAP1 and cIAP2, and Fas-Associated protein with Death Domain (FADD) are recruited to form a DISC that activates caspase-8 [5]. In the intrinsic (mitochondrial) pathway of apoptosis (see Figure 1), death inducing stimuli activate pro-apoptotic Bcl-2 family proteins to alter mitochondrial membrane permeability and induce cytochrome c release from mitochondria [6]. Cytosolic cytochrome c promotes the assembly of an apoptosome, a multimeric protein complex containing Apaf-1 and cytochrome c [7,8]. The apoptosome recruits and activates initiator caspase-9, which then cleaves executioner caspase-3 or -7 [9].

For a long period of time, caspases have been predominantly studied for their pro-apoptotic functions. However, functional studies of caspases in recent years have changed this view. It is increasingly clear that caspases have non-apoptotic functions in multiple cellular processes, such as inflammation, cell differentiation and proliferation [10]. In the nervous system, caspases have been shown to play a non-apoptotic role in synaptic plasticity [11,12], dendritic pruning during development in Drosophila neurons [13,14], chemotropic responses of retinal growth cones in Xenopus [15], neurite outgrowth [16], and the development and maturation of olfactory sensory neurons [17]. This review will focus on the functions of caspases in modulating synaptic transmission under both physiological and pathological conditions, and its relevance to cognition.

### Mitochondrial apoptotic pathway and caspase-3 in LTD

Synaptic plasticity, the ability of synapses to adjust their strength, is an important means by which the nervous system responds to prior experience and adapts to environmental changes. The change in synaptic strength can be transient (seconds to minutes) or last for prolonged period of time. Long-lasting forms of synaptic plasticity play a crucial role in the refinement of neuronal connections during development and in cognitive functions such as learning and memory [18,19]. In the mammalian brain, NMDA receptor-dependent long-term potentiation (LTP) and long-term depression (LTD) of synaptic transmission are two major forms of long-lasting synaptic plasticity. The movement of AMPA receptors into and out of the synapse appears to be the primary cell biological mechanism underlying the change of synaptic efficacy during LTP and LTD. However, the signaling pathways and molecular mechanisms underlying LTP and LTD are not clearly understood.

One interesting feature of synaptic plasticity is the morphological change that accompanies functional modification of the synapse. LTP is associated with formation and growth of dendritic spines [20-23] whereas LTD is associated with shrinkage and loss of spines [23-25]. We hypothesized that LTP and LTD reflect opposing cell biological processes that control cellular growth. Could the mediators of apoptosis - which represents the major pathway for controlled cellular involution - also play a role in the weakening and elimination of synapses?

**Figure 2:**
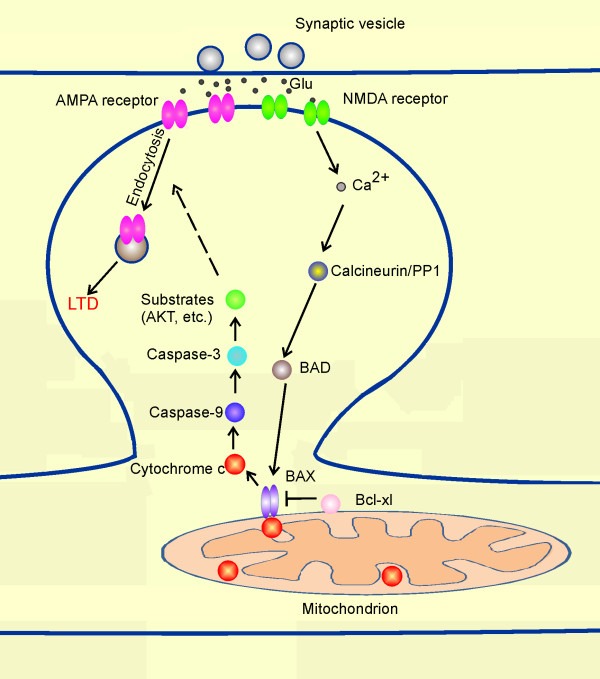
**Involvement of mitochondria and intrinsic apoptotic pathway in LTD**. The mitochondrial pathway is required for NMDA receptor-dependent long-term depression of synaptic transmission. NMDA receptor activation leads to moderate activation of the mitochondrial pathway, which is required for AMPA receptor endocytosis.

We recently reported that caspase-3 and the mitochondrial pathway of apoptosis play a critical role in LTD induction [11]. In caspase-3 knockout mice, NMDA-receptor dependent LTD in CA1 neurons is abolished, whereas LTP can be induced normally. LTD is also specifically blocked by pharmacologic inhibition of caspase-3 (the executioner caspase) and caspase-9 (the initiator caspase upstream of caspase-3 in the intrinsic pathway of apoptosis), but is unaffected by a caspase-1 inhibitor [11]. In LTD, caspase-9 and caspase-3 are activated through the engagement of the intrinsic pathway by activation of BAD, which is a pro-apoptotic Bcl-2 family protein [26] (see Figure 2). BAD is also activated in apoptotic cells, but in neurons undergoing LTD, BAD is activated to a lower level and transiently, which leads to a moderate degree of caspase activation that is sufficient to promote AMPA receptor internalization [26]. More prolonged and much higher levels of caspase-3 activation is required to induce cell death [26], and AMPA receptor endocytosis may contribute to apoptotic signaling [27]. Active caspase-3 in neurons undergoing LTD is required for AMPA receptor endocytosis without causing cell death [11]. In caspase-3 knockout mice and BAD knockout mice that have deficient NMDA receptor-induced caspase-3 activation, AMPA receptor internalization is blocked [11,26]. Similarly, BAD and BAX KO mice are defective for LTD [26]. LTD is also blocked by overexpression of Bcl-xl (an anti-apoptotic member of the Bcl2 family) or XIAP (a direct protein inhibitor of caspase-9 and caspase-3) [11] (see Figure 1). In contrast, LTP is unaffected by pharmacologic, genetic or molecular disruption of caspase-3 and the intrinsic apoptotic pathway [11,26,28]. Together these findings confirm the importance of the mitochondrial apoptotic pathway - which culminates in activation of caspase-3 - specifically in the induction LTD.

The mitochondrial apoptotic pathway is engaged by the protein phosphatase calcineurin and PP1, which are activated downstream of NMDA receptors [19]. Active calcineurin and PP1 dephosphorylate BAD to initiate the mitochondrial apoptotic pathway in neurons [26,29]. The critical involvement of calcineurin/PP1 in the intrinsic pathway of apoptosis can explain why these protein phosphatases are necessary for LTD induction, although this does not exclude the possibility that calcineurin/PP1 also act on other relevant substrates in LTD (such as AMPA receptors, PSD-95) [19,30].

Active caspases are presumed to function in LTD as proteolytic enzymes. Crucial steps in understanding the mechanism of caspases are to identify the substrates of caspase-3 that are relevant to LTD, and to figure out how cleavage of these substrates leads to AMPA receptor internalization. The pro-survival and pro-LTP protein kinase Akt (a known substrate of caspase-3 in cell death) is thought to play a role in suppression of LTD by phosphorylating and inhibiting GSK3β [31]. Overexpression of a mutant form of Akt that is not susceptible to cleavage by caspase-3 prevents the induction of LTD, consistent with the idea that Akt proteolysis by caspase-3 is required for LTD in neurons [11]. Thus the mitochondria-caspase cascade can fit into the key signaling pathways for LTD induction.

LTD is typically regarded as "homosynaptic" (occurring only at stimulated synapses), though in some conditions, it can also be observed at un-stimulated synapses ("heterosynaptic LTD") [32]. We postulate that only mitochondria at or near the activated synapses take part in LTD induction. Because LTD stimulation results in only moderate and transient activation of the mitochondrial apoptotic pathway, activated caspases are restricted to the vicinity of stimulated synapses, thereby preventing long-distance spread of LTD. Although the spatial and temporal properties of mitochondrial activation in LTD are still unclear, we found that mitochondria can be very close to synapses (some are even present in dendritic spines), and their morphology, distribution and motility are regulated by synaptic stimulation [33].

### Caspases in LTP and learning and memory

Caspase-1 (interleukin-1β-converting enzyme, ICE) is the first identified mammalian member of the caspase family [2]. Active caspase-1 proteolytically processes cytokines IL-1β and IL-18, and is a well-known pro-inflammatory caspase [1]. In addition to its pro-inflammatory function, active caspase-1 has been shown to inhibit LTP. A peptide caspase-1 inhibitor (z-YVAD-FMK) enhances NMDA receptor-dependent LTP when applied to hippocampal slices [12]. The effect of caspase-1 inhibitor on LTP could be mediated by its substrate IL-1β, because application of IL-1β to hippocampal slices inhibits LTP in CA1 [34], CA3 [35] and dentate gyrus [36-38], and intracerebroventricular injection of IL-1β into rats impairs LTP in dentate gyrus [39,40]. However, there is no published evidence from caspase-1 or IL-1β KO mice that this pathway is involved in the control of synaptic plasticity.

Synaptic plasticity is crucial for cognitive functions of the brain, such as learning and memory. Consistent with their functions in LTD and LTP, caspase-3 and caspase-1 have been reported to contribute to learning and memory. In the zebra finch auditory forebrain, caspase-3 activity is necessary for memory consolidation during birdsong learning, and novel-song exposure causes a rapid and transient release of active caspase-3 from its inhibitory protein XIAP into dendritic spines [41]. Chronic brain infusion of caspase-1 inhibitor in aged rats ameliorates age-related increase in hippocampal IL-1β, and improves hippocampus-dependent contextual memory [42]. The above pharmacologic experiments suffer from the caveat of drug specificity. Behavioral studies of caspase-3 and caspase-1 knockout mice have not been reported yet.

### Caspases in neurodegeneration

Progressive decline in cognitive abilities is the prominent symptom of Alzheimer's disease (AD). Synapse loss and reduction of synaptic proteins are detected in the hippocampus and cortex at early stages of AD, and correlate with cognitive dysfunctions [43]. Synaptic pathology is believed to be a major contributor to learning and memory impairment in AD, but its etiology remains unclear. Active caspase-6, an effector caspase, has been reported in post mortem brains of prodromal AD patients who do not yet display apoptotic morphology, and the level of caspase-6-cleaved Tau inversely correlates with the global cognitive score [44]. The notion that caspase activity might have detrimental non-apoptotic effects on synapses in early AD is supported by recent studies in transgenic mouse models of AD. In Tg2576 transgenic mice that express mutant human amyloid precursor protein, caspase-3 activity is enhanced in dendritic spines roughly coincident with the onset of memory decline and in the apparent absence of neuronal cell death in these animals [45]. In Tg4510 Tau transgenic mice, which develop neurofibrillary tangles, active effector caspases are detected in some neurons preceding the appearance of tangles [46]. Active effector caspases appear to initiate tangle formation by cleaving tau, but do not induce cell death [46]. Analysis of synaptic functions in the Tg2576 mice suggests that caspase activation leads to dephosphorylation and removal of AMPA receptor subunit GluA1 from synapses (possibly by activating calcineurin), altered glutamatergic synaptic transmission, and enhanced LTD in hippocampal CA1 neurons [45].

Impaired synaptic plasticity is also implicated in the pathophysiology of AD. In vitro, amyloid β1-42 (Aβ1-42) applied for two hours to hippocampal slices inhibits LTP; this Aβ-mediated inhibition depends on the mitochondrial pathway of apoptosis, caspase-3, cleavage of AKT by caspases, as well as GSK3β and BAX [28,47]. The impairment of LTP by Aβ requires NMDA receptor function, and conversely, Aβ can enhance LTD [28,48]. However, unlike LTD, caspase-9 inhibitor does not block the effect of Aβ on LTP [47], suggesting that Aβ can activate caspase-3 through both mitochondria-dependent and mitochondria-independent pathways. Thus Aβ suppression of LTP involves - at least in part -- the same mechanisms as used in LTD. These studies suggest that abnormally active caspases may contribute to synaptic deficits in the AD brain, even before frank neuronal death. It is intriguing that injection of caspase-3 inhibitor into the Tg2576 transgenic mice ameliorates the memory defects [45].

## Conclusion

The discovery of caspases as amplifiers and executioners of apoptosis highlighted their important role in programmed cell death. Until recently, the presence of active caspases was believed to lead irreversibly to cell death, and thus was a widely used marker of apoptotic cells. However, active caspases can be also detected in cells that are not destined to die, and it is now widely accepted that caspases can play non-apoptotic roles in various developmental and physiological contexts. In the past few years, studies from several groups collectively point to an essential function of caspases in synaptic plasticity, independent of neuronal cell death. Two initiator caspases (caspase-1 and caspase-9) and the effector caspase-3 are shown to regulate long lasting synaptic plasticity in hippocampal neurons. In particular, there is compelling evidence that the induction of NMDA receptor-dependent LTD is critically dependent on caspase-3 activation, and moreover, active caspase-3 is sufficient to induce synaptic depression. BAD and BAX induced mitochondrial release of cytochrome c plays a crucial role in activating caspase-3 in LTD. Active caspase-3 is required for AMPA receptor endocytosis and consequent reduction of synaptic strength, but the mechanism of how caspase-3 controls AMPA receptor trafficking is still unclear.

Emerging evidence indicates that caspases are active in early stages of AD, and could mediate synapse dysfunction and loss before the advent of cell death and neurodegeneration. These new insights have potential implications for the treatment of AD, and they highlight the regulatory role of caspases in synaptic plasticity under both normal and pathological conditions.

## Competing interests

Morgan Sheng is an employee of Genentech Inc.

## Authors' contributions

ZL and MS wrote the manuscript. All authors read and approved the final manuscript.
